# Dementia prevention through the eyes of individuals at risk: insights from a satisfaction survey within the programme for dementia prevention in Luxembourg

**DOI:** 10.3389/fragi.2026.1712500

**Published:** 2026-01-16

**Authors:** Dorothee Erz, Valerie E. Schröder, Amna Skrozić, João M. Loureiro, Nina Possemis, Rejko Krüger

**Affiliations:** 1 Department of Neurology, Centre Hospitalier de Luxembourg (CHL), Luxembourg, Luxembourg; 2 Transversal Translational Medicine, Luxembourg Institute of Health (LIH), Strassen, Luxembourg; 3 Luxembourg Centre for Systems Biomedicine (LCSB), University of Luxembourg, Esch-sur-Alzette, Luxembourg

**Keywords:** cognitive complaints, cognitive impairment, dementia, personalised prevention, programme dementia prevention, satisfaction survey

## Abstract

**Introduction:**

Establishing a nationwide prevention programme can be challenging, particularly in the field of dementia, as role model projects are lacking, and the topic is still associated with stigma. Hence, a satisfaction survey for the programme dementia prevention (*pdp*) in Luxembourg was conducted to obtain direct feedback from the participants on how the programme and its multidomain interventions are accepted and evaluated.

**Methods:**

In 2023 and 2024, a satisfaction survey was sent out to all eligible *pdp* participants (n = 575). Participation was voluntary and anonymous, and the survey could be completed in paper form or online. The questionnaire contained 12 closed-ended questions rated on a Likert-scale and 3 open questions.

**Results:**

302 (52.5%) participants returned the survey. The analysis revealed a high level of satisfaction with an overall average satisfaction of 4.7/5 points (SD = 0.6). 281 participants (95.9%) would recommend the *pdp* to others. A breakdown of the statements provided insights into areas of interest from the participants’ perspective.

**Discussion:**

Our study shows that the *pdp* is well received by its participants, underlined by a high level of satisfaction and positive responses to what *pdp* offers. Findings showed that interactions were perceived as informative to gain insights into one’s own cognitive performance and raised awareness of possibilities to reduce dementia risk, i.e., via lifestyle changes. Our results can serve as orientation for the implementation of emerging prevention programmes in different healthcare settings.

## 1 Introduction

Based on the increase of neurodegenerative diseases, the number of individuals affected by dementia is expected to reach 152.8 million by 2050 (Nichols et al., 2022). As pharmaceutical interventions remain inaccessible to a large number of people, there is a need to relieve both the social burden caused by the disease itself as well as the burden on the healthcare system associated with dementia care (World Health Organization, 2021). Dementia prevention has gained traction since various studies have demonstrated a substantial effect of preventative measures on dementia risk factors (Bartlett et al., 2023; Ngandu et al., 2015). In the Lancet commission update of 2024, Livingston and colleagues extend the number of modifiable dementia related risk factors to a total of 14, namely, less education, hearing loss, high levels of LDL-cholesterol, depression, traumatic brain injury, physical inactivity, smoking, diabetes, hypertension, obesity, excessive alcohol consumption, social isolation, air pollution, and untreated vision loss. The authors state that these account for up to 45% of all dementia cases on a population level (Livingston et al., 2024). Targeting those modifiable risk factors through specific health changes creates a window of opportunity to reduce, or at least slow down, further cognitive decline (Solomon et al., 2021). Hence, the aim of dementia prevention initiatives is to raise awareness about evidence-based dementia-related risk factors in the population to reduce dementia risk and to support brain health. Dementia prevention measures are expected to be most efficient in people at risk, including individuals with subjective and/or objective cognitive complaints (Frisoni et al., 2023; Pozzi et al., 2024). With growing knowledge of these early stages of neurodegenerative diseases, healthcare is beginning to shift towards at-risk individuals to influence progression already on an early stage (Pozzi et al., 2024). This transformation leads to new requirements on the healthcare landscape. There is a need for public centers dedicated to individualised prevention of dementia risk, by providing services such as risk assessment, risk communication and personalised prevention (Frisoni et al., 2023; Pozzi et al., 2024). Such an approach not only contributes to increasing health literacy in the context of dementia, but also supports at-risk individuals in proactively addressing their own modifiable risk factors and to take self-empowered decisions about their future in case of disease progression.

The Finish Geriatric Intervention Study to Prevent Cognitive Impairment and Disability (FINGER) was the first to introduce multi-domain lifestyle interventions as a measure of dementia prevention and was able to successfully provide evidence for positive effects on cognitive performance and general health (Kivipelto et al., 2018; Kivipelto et al., 2020; Ngandu et al., 2015). Since then, various studies have confirmed that dementia prevention can be an effective tool in reducing dementia risk. The Australian ISLAND study (Bartlett et al., 2023) provided their participants (N = 7,264) personalised feedback on their individual risk factor profile. Participants were offered the option to learn more about dementia risk reduction with the help of an online educational programme targeting management of health risk behaviour. 58.8% of the participants used the opportunity to learn more about preventing dementia in an online course. For these participants, an improvement of 26% in the risk factor status was observed over 3 years. In 2018, Schiepers and colleagues introduced a prediction model for late-life risk of dementia, based on the LIfestyle for BRAin health (LIBRA). They report that a one-point increase in LIBRA scores translates into a 19% higher risk for dementia and a 9% higher risk for cognitive impairment (Schiepers et al., 2018). Furthermore, a Cochrane Intervention Review showed high-certainty evidence of the positive impact of multi-domain interventions, including cognitive training, on cognitive functioning (Hafdi et al., 2021). Hafdi and colleagues outline the relevance of even very modest effects on preventive implications in a population-based setting, especially when they are sustainable.

In order to enhance dementia prevention, the nationwide integrated care concept “programme dementia prevention (*pdp*)” was established in Luxembourg in 2018 (Schröder et al., 2024). Since then, the programme has been funded by the Ministry of Health in Luxembourg and is open to all people exhibiting self- or externally-reported cognitive complaints who get referred by their treating physician and are at least 18 years old. The baseline visit of the *pdp* is separated in a first appointment with a duration of 2 to 3 h, which includes detailed explanations about the aim and organisational aspects of the programme. This is followed by an anamnesis and comprehensive neuropsychological assessment containing standardised tools evaluating different cognitive functions, such as memory, attention, executive functions, visuo-spatial abilities and language. Participants are informed that a break can take place at any time and that the appointment can be split into two appointments, if necessary. In a second appointment, the results of this assessment are explained orally to the participant. Taking into account age, sex, and educational level, participants are classified as subjective cognitive decline (SCD) or mild cognitive impairment (MCI). In case they present neither subjective cognitive complaints nor objectifiable cognitive deficits, they are classified as “no subjective/objective cognitive impairment”. Participants who show a more advanced cognitive decline with associated loss of autonomy in activities of daily living are classified as “suspicion of dementia” and are referred to their respective treating physician for consolidating the diagnosis. Furthermore, they are informed about specialised institutions for people living with dementia. For the discussion of the dementia-related risk factors, the *pdp* team establishes for each participant an individual risk profile based on the risk factors identified by Livingston and colleagues (2020) and Deckers (2017). In line with their personal profile, participants are recommended a tailor-made set of “vouchers” that allow the participation in various preventive measures to reduce their personal dementia risk. National partners of the *pdp* offer a broad spectrum of these multi-domain measures, including cognitive training, physical and/or social activities, dietary counseling or psychological counseling, distributed in the country and available in multiple languages such as Luxembourgish, French, German and English. Participation in the *pdp* as well as the voucher activities are voluntary and free of charge. At the end, both the participant and the referring physician receive a written report of the test results. After their completed baseline visit, participants are invited to attend regular follow-up visits.

Studies from various healthcare disciplines have shown that patients’ experiences are positively associated with clinical effectiveness, health outcomes, adherence to treatments and preventative care (Doyle et al., 2013). Furthermore, patients’ experiences can serve as an indicator of the quality of care (Anhang Price et al., 2014; Kingsley and Patel, 2017). Surveys are a commonly used tool to reflect on patient-reported benefits, patient-reported experiences and their satisfaction (Friedel et al., 2023). Accordingly, collecting patient experience data can be the first step in improving the quality of health care (Ahmed et al., 2014). The knowledge that emerges from a satisfaction survey can improve communication, uncover overlooked problems and ensure a more personalised care in the healthcare system (Friedel et al., 2023). However, little is known about the experiences and expectations of participants on integrated care in the context of dementia prevention. As outcomes of prevention programmes such as the *pdp* are challenging to measure, due to multiple confounding variables, participants’ experiences can serve as one pillar for evaluating such a programme. Therefore, the aim of our satisfaction survey is to systematically collect the experiences and satisfaction of at-risk individuals with their participation in the *pdp*. In addition, questions on content-related aspects of programme should allow conclusions to be drawn about the performance of the programme and its offers. The findings of this study are intended to shed light on the participants’ perspective on *pdp*. On another level, the feedback from participants shall be used to boost the programme’s performance and enhance adherence by best addressing participants’ needs.

## 2 Methods

### 2.1 Participants and sample attrition

**FIGURE 1:**
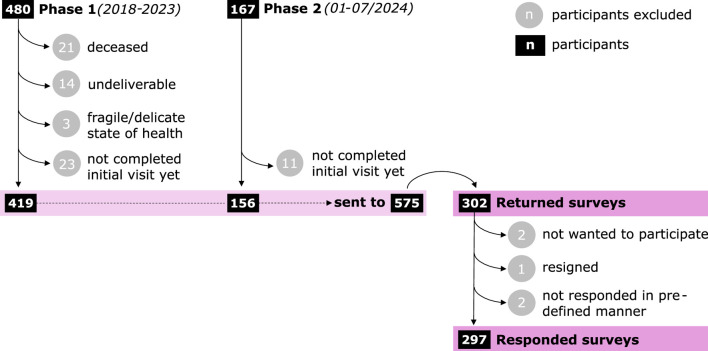
Flowchart illustrating sample attrition and response frequency.

A total number of 575 surveys were sent out in two phases, as illustrated in Figure 1. Phase 1 included all participants with a *pdp* visit between 2018 and 2023. The aim of Phase 1 was to retrospectively collect feedback on previous years, regardless of time intervales between participants’ baseline/follow up visits and timing of the survey. Phase 2 included all participants with a baseline visit between January and July 2024. The purpose of Phase 2 was to ensure a timely data collection, as the maximum interval between the second appointment of the baseline visit and receipt of the questionnaire was 1 month.

Participants who had not completed their baseline visit before the endpoint of one Phase were excluded from the survey. For these participants, the second appointment for the communication of the neuropsychological test results as well as the risk factor assessment and the voucher distribution was still pending, but this session is important for completing the questionnaire.

### 2.2 Ethics

The programme was approved by the National Research Ethics Committee (Comité National d'Ethique de Recherche (CNER) Ref: 201511/01). All participants provided written informed consent before participating in the programme. The study was conducted in accordance with the 1964 Helsinki Declaration and its later amendments.

### 2.3 Material

The survey was designed by the *pdp* team and provided in German, French or English to the participants. It was composed of a qualitative as well as a quantitative part (see Supplementary Material). To maximise the response rate, the hurdles were kept as low as possible by offering a short survey with a completion time of approximately 10 min and a mixed modality for completion (online and mail) (Booker et al., 2021; Story and Tait, 2019). The survey was adapted to the cognitive difficulties often present in the participating population of the *pdp*. Accordingly, the survey was designed to be comprehensible, short and easy to answer. Furthermore, a picture of the clinical *pdp* team was included on the bottom of the survey letter to facilitate the recognition of the team of the *pdp*. Respondents were asked to rate their satisfaction by answering 12 closed-ended questions using a 5-point Likert scale, with the following designations: 1-“strongly disagree”; 2-“somewhat disagree”; 3-“neutral”; 4-“somewhat agree”; 5-“strongly agree”. The closed-ended questions covered the following topics: team; organisation and communication; experience on site; benefits of the programme, and general satisfaction. The qualitative part, consisting of three open-ended questions, was included to encourage the participants to share more detailed and personal information about their experience without being determined by the researcher’s presuppositions (Allen, 2017). The free-text format allowed participants to share what they liked about the programme, where they still see room for improvement and an anecdote regarding their participation.

Following the evaluation of Phase 1, the second open question of the qualitative part was modified for Phase 2. The answers to this second question showed that the wording of the question was not precise enough for some participants, as they answered the question in terms of room for individual improvement rather than improvements to the programme (see survey in Supplementary Material).

### 2.4 Design

The study used a within-subject design, meaning that all 575 participants were sent a two-sided single-sheet questionnaire along with a pre-addressed and stamped return envelope. Participants received the survey in their preferred language as marked in the *pdp* system. They were asked to rate their experience of participating in the *pdp* by either completing the survey in paper form, with a prepaid postage, or using a QR-code on the survey letter. The QR-code led to the online questionnaire, which was generated using SoSci Survey (Leiner, 2024) and was made available to users via www.soscisurvey.de. All surveys were given a random four-digit number, which had to be entered when completing the survey online. The purpose of this coding system was to avoid duplicates and ensure the anonymity of the survey. Furthermore, the first digit of the four-digit code indicated the participant’s time of participation (see Table 1). No reminders or incentives were given.

**TABLE 1:** Response rates per survey phase.

Survey phase	Number of surveys sent out	Number of returned surveys	Response rate
Total	575	302	52.5%
Phase 1	419	205	48.9%
Code 1	269	115	42.8%
Code 2	150	90	60.0%
Phase 2	156	97	62.2%

Phase 1 = *pdp* visits between 2018 and 2023 (regardless of whether baseline or follow-up visit); Code 1 = *pdp* visits between 2018 and 2022; Code 2 = *pdp* visits in 2023; Phase 2 = *pdp* baseline visits between January and July 2024.

### 2.5 Data curation

By using the aforementionded four-digit number to remove duplicates from the data, two participants who completed the questionnaire both online and on paper were detected. In addition, one incomplete data entry was deleted as the same participant made a new complete data entry right afterwards. As illustrated in Figure 1, five out of 302 returned surveys had to be excluded resulting in a total number of 297 correctly completed surveys qualifying for further statistical analyses.

### 2.6 Data analysis

Data analysis of the quantitative data was conducted in R (Version 4.4.2) and R studio (Version 2024.12.0.467) (Posit team, 2024; R Core Team, 2024). The data was processed using the R packages dplyr (Wickham et al., 2019) and car (Fox and Weisberg, 2019). A Mann-Whitney-U-Test was used due to the ordinal nature of the Likert-scale data and a non-normal distribution of assumptions and allowed to investigate whether there was a difference in satisfaction related to the time point of the participation (Phase 1 vs. Phase 2). For the qualitative data analysis, a breakdown of the (sub-) topics mentioned by the participants in the open-ended question part should provide insights into the most relevant topics from the participants’ perspective. Therefore, a conventional content analysis was conducted, coding all comments in a corresponding theme (Hsieh and Shannon, 2005). Themes and subthemes were conducted inductively and confirmed by peer feedback.

## 3 Results

### 3.1 Descriptive data

Out of 575 surveys sent out, a total of 302 were returned, which corresponds to a response rate of 52.5%. Differences in response rates per data collection phase are presented in Table 1. A total of 266 respondents (88.1%) returned their feedback by post; 36 (11.9%) participants used the online format. Most participants replied in German (56.3%), with French being the second most returned survey language (42.1%). Only five individuals (1.7%) replied in English.

### 3.2 Quantitative data

The response frequency for all 12 questions is shown in Table 2. The mean of the overall satisfaction as calculated from all questions was M = 4.7 points of 5 points with a standard deviation (SD) of 0.6 points. With mean values of 4.9 points, questions 1 (‘Friendly team’), 2 (‘Professionalism’) and 6 (‘Waiting time on site’) received the highest level of approval. Among the respondents, 281 (95.9%) participants would recommend the *pdp* to others. Across all questions, the proportion of missing data was highest for question 11 (‘Benefit of the programme’), with 11 (3.7%) respondents having left this question out.

**TABLE 2:** Response frequency on closed-ended questions.

Questions	n	1 (%)	2 (%)	3 (%)	4 (%)	5 (%)	*M*	*SD*
1. The team was friendly.	297	0.7	0.0	1.0	3.7	94.6	4.9	0.4
2. The team was professional.	294	0.7	0.0	0.3	9.9	89.1	4.9	0.5
3. I felt comfortable.	293	0.7	0.0	1.4	11.9	86.0	4.8	0.5
4. The waiting time for an appointment was appropriate.	292	1.4	1.7	4.5	23.6	68.8	4.6	0.8
5. I was satisfied with the communication and the organisation of the appointments by the administrative office.	295	1.0	0.0	1.0	11.5	86.4	4.8	0.5
6. The waiting time on site was acceptable.	294	0.7	0.3	0.7	7.8	90.5	4.9	0.5
7. The length of the neuropsychological testing was appropriate.	294	0.7	1.0	4.8	19.4	74.1	4.7	0.7
8. The participation helped me gain a better understanding of my cognitive performance (e.g., memory, language, concentration, …).	291	1.0	1.7	8.2	26.8	62.2	4.5	0.8
9. I found the discussion of my risk factors helpful.	292	0.7	1.7	8.9	25.7	63.0	4.5	0.8
10. I was satisfied with the time devoted to me in the *pdp*.	295	0.7	0.7	2.0	14.9	81.7	4.8	0.6
11. I have benefited from the participation.	286	0.7	0.7	7.3	19.6	71.7	4.6	0.7
12. I would recommend the programme to others.	293	1.0	0.3	2.7	12.3	83.6	4.8	0.6

Response frequency in percentages. n = number of valid responses; 1-“strongly disagree”, 2-“somewhat disagree”, 3-“neutral”, 4-“somewhat agree”, 5-“strongly agree”.

### 3.3 Participants’ experience vs. perceived benefit

All questions were categorised in questions about “experience” or questions about “perceived outcomes” to better compare these two conceptual groups (see Table 3).

**TABLE 3:** Participants’ satisfaction grouped by experience-related and benefit-related questions.

Question category	Question number	Level of satisfaction		
		-	0	+
Experience	1, 2, 3, 4, 5, 6, 7, 10, 12	1.3%	2.0%	96.7%
Perceived benefit	8, 9, 11	2.2%	8.2%	89.6%

### 3.4 Group comparison

A Mann-Whitney-U-Test showed that the overall mean was significantly (*p* < 0.001) higher for Phase 2 (M = 4.79) than for Phase 1 (M = 4.68). However, after performing a Mann-Whitney-U-Test comparing each question, no significant mean differences were found between the two phases after Bonferroni correction for multiple testing was applied.

### 3.5 Qualitative data

**FIGURE 2:**
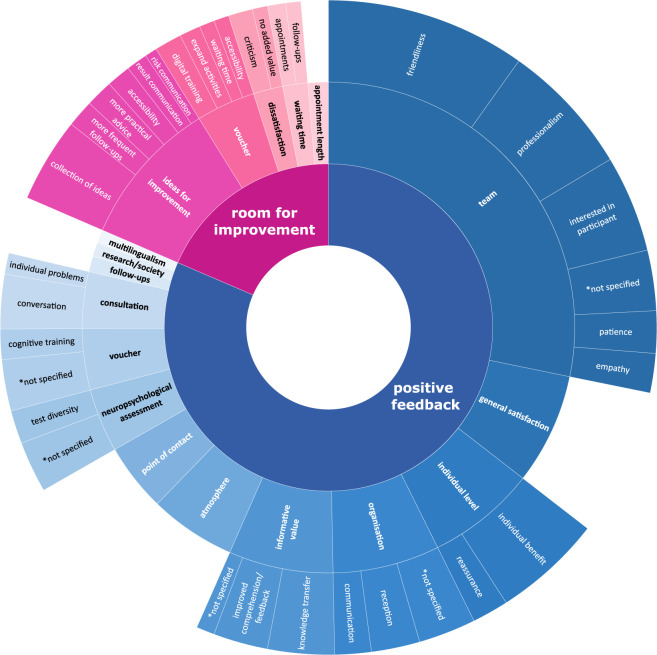
Frequency of open-ended responses grouped by (sub-) themes. *not specified = different statements that did not result in a specific subtheme; A detailed description of subthemes can be found in paragraph “Subthemes in free text comments”.

66.7% (n = 198) of respondents provided a total number of 419 free text answers. This high number of responses to the open-ended questions, where each respondent could give a maximum of three answers, reflect a high level of commitment. 82 comments were not considered for further analysis as they had no informative value (i.e., one-word answers, such as “none”). Following an inductive approach for qualitative data analysis, a breakdown of the topics mentioned by the participants in the open-ended questions provided insights into the main topics from the participants’ perspective. To create meaningful subthemes, a minimum number of four thematically related statements was defined. Comments that addressed several subtopics were coded multiple times. 546 aspects accounted for positive topics, whereas 124 aspects were assigned to room for improvement, resulting in one-fifth of the feedback proposing further improvements to the programme. In Figure 2, all topics and subtopics according to the frequency of occurrence in the comments are illustrated.

### 3.6 Subthemes in free text comments

Although all statements of a topic are thematically related, the different subtopics allow to further identify underlying aspects of that specific topic. For this reason, some of the subtopics are described in more detail to better capture the scope of the various aspects and their relevance for *pdp* participants.

The team was most frequently mentioned positively in the comments, emphasising that participants consider the positive characteristics and skills of the team to be particularly important and that these contributed strongly to their satisfaction. This is linked to the atmosphere of the programme, which was described as comfortable and relaxed and the environment was perceived as being reassuring. Participants reported feeling safe, respected and supported when being on site. One participant stated: “I had the impression that the objective of these people was to make me feel at ease.”

The service of the *pdp* was described as an important contact point and participants appreciated having a contact person. The support through the *pdp* in general and the existence of the programme was highlighted in comments like: “Knowing that someone cares about my concerns.”; “It's great to have the opportunity to be part of such a programme.”; “The fact that an institution, such as yours, is proactively dealing with a concern that worries many elderly people.”; “I just want to express my gratitude for creating such a programme. I’m very concerned about this subject and it's wonderful that governments are putting things like the *pdp* in place.” While another participant suggested: “Perhaps the conversations could be more intensive and more frequent.”

The topic organisation mainly comprised the administrative part of the programme. This ranged from initial telephone contact with participants to the organisation of appointments and information on documents and questionnaires. One participant acknowledged: “They remind you of your appointment in advance.”

The neuropsychological assessment is conducted during the first appointment of the baseline visit of the *pdp* following an anamnesis. Participants appreciated that they received a written report and feedback related to their cognitive performance. One participant liked: “That the tests have given an overview, where you can see what you are good at and what you are weak at.” Whereas another participant suggested: “If possible, shorten the tests or do them in 2 sessions.”

During the second appointment, the neuropsychologist explains to the participant the neuropsychological test results and the risk factor profile using the LIBRA index. In this session, participants receive personalised information on cognition as well as on dementia-related modifiable risk factors. Comments described that the participation was of informative value to the participants and that they appreciated the explanations, education, counseling, and tips they received. The simplicity of the knowledge transfer was positively emphasised: “I’d like to thank your team for listening to me, helping me to better understand my cognitive performance and found the discussion about my risk factors useful.”; “The *pdp* helped me put this diagnosis into perspective and showed me that, despite some memory problems, there are ways of slowing down or avoiding the path I had already imagined!”; “I was able to learn what possibilities there actually are to train your fitness.” Some participants offered ideas to improve the knowledge transfer, such as: “Perhaps give out a brochure (or a list of books) on what we can do to prevent dementia.”

Participants are encouraged to return for follow-up visits in a regular interval. They value this offer, as one participant put it: “I hope to be able to take part for a long time to come.” or “I always look forward to the next appointment!”. Other participants proposed, an improvement: “could be in the area of tracking of the patients a bit more” or “It would be nice if these tests could be repeated at shorter intervals (1/2 year?). Perhaps less detailed, but more often.”

Some participants emphasised positively that the *pdp*, on a metalevel, adds value for both research and the community. The commitment of some participants to support research was demonstrated by the willingness to participate in subject related studies in the past. A participant stated: “This prevention is of great benefit to society as a whole (family members, employers, etc.).”

As Luxembourg is a multilingual country, language plays a key role in the wellbeing of the participants and multilingualism was appreciated. However, the fact that the national language ‘Luxembourgish’ is also spoken was emphasised positively.

Concerning the accessibility, participants reported on the inconvenient parking situation as well as the need for improved accessibility by public transport. This issue was also noted in relation to the voucher activities. This was linked to the request for an expansion of the voucher partners in order to be able to take advantage of more interventions in the neighborhood. However, it was not only a geographically denser network of activities that was desired, but also an expansion of the offer types.

One intervention offered by the *pdp* is a license for a digital cognitive training. Although this option has received plenty of positive feedback, some participants see room for improvement in terms of the range of exercises as well as the introduction to the application and its use.

In the section room for improvement, a collection of individuals ideas was compiled. For instance, participants asked for shorter waiting times for reports, a more digital approach and improved visualisation of their cognitive results and their risk factors over time.

## 4 Discussion

The purpose of this study was to gain insights into the perspective of at-risk individuals regarding their experiences and opinions participating in the nationwide dementia prevention programme in Luxembourg. Our findings not only provide valuable information about the reception of this first nationwide dementia prevention programme, but will also help to further adapt the programme to the needs of at-risk individuals for dementia. Both the qualitative and the quantitative results underscore the participants’ high appreciation for the *pdp* and great esteem for the team as well as recognition of their professionalism and an appraisal for operating procedures. The data underlines that the concept of the programme is well received by the participants but might be improved by expanding the types and locations of interventional activities as well as providing more in-depth education on risk communication and an adaptation of the dementia prevention counseling. The continued recruitment success of the *pdp* shows that the implementation of a dementia prevention programme is feasible. Moreover, this study demonstrates that the programme receives a high level of acceptance and satisfaction among participants.

As response rates are highly depending on the data-collection medium, the length of the survey, the fact whether reminders or incentives were given, and the interviewee type, there is no consensus on what an ideal or average response rate is (Booker et al., 2021; Meyer et al., 2022). However, the achieved response rate of 52.5% can be considered adequate for mail and online surveys in literature (Story and Tait, 2019). A study led by Krist et al. (2014) showed that over all administration approaches, an average response rate of 49.6% was achieved. However, when the survey was completed by the patients themselves without help of staff, the response rate decreased to 30.1%. In our survey, a large discrepancy can be observed in the choice of response modality, as the digital version was only used to a small extent. This may be explained by the average age of *pdp* participants of 68.3 years in the programme and an assumed lack of technical skills to scan a QR-code. For future studies, a web-based modality with an invitation email referring to the survey via a hyperlink would be recommended because this may increase the response rate while keeping the technical complexity to a minimum. Furthermore, as Luxembourg is a multilingual country, the results show that it is worth offering the survey in several languages. The Portuguese community represents the largest minority in Luxembourg, accounting for 13.1% of the total population (Statistiques.lu, 2025). Therefore, we will offer a Portuguese version of the survey in the future, to be more inclusive and to increase the response rate. The sub-analysis of our data on the response rate suggests that the response rate increases when the time interval between visit and survey was shorter. Considering that no incentives, no assistance from healthcare professionals or reminders were given, the response rate of the satisfaction survey can be considered moderate to high compared to other surveys in the primary care setting (Ahmed et al., 2014; Booker et al., 2021).

The significant increase in satisfaction (0.11 points) between the two data collection periods (Phase 1 vs. Phase 2) can be explained by the ongoing nature of the programme and the resulting adjustments made to continuously improve the *pdp*. For instance, participants in Phase 1 indicated that they wished for a French version of the digital cognitive training. This version was already available to the participants who were asked for their feedback in Phase 2 of the survey. The same applies for the number of appointments, which was reduced from three to two due to workflow adjustments in the early stages of the programme. Therefore, the duration of a visit for some respondents in Phase 1 who participated in the initial phase of the programme may have differed from the duration for respondents in Phase 2. Respondents who participated in the initial phase of the programme could not yet have benefited from adaptations of interventions or workflow adjustments that were incorporated into the programme over time. This specific finding underlines that participants value how the *pdp* is evolving and that the adaptations in the programme are perceived as improvements in the perspective of participants.

The aim of the *pdp* is to reduce the individual risk for developing dementia by raising awareness among participants for specific dementia-related risk factors. In the “National Poll on Healthy Aging (NPHA)” by the University of Michigan, 44% of participants reported being worried about developing dementia, however, only 5% indicated that they ever discussed ways to prevent dementia with a physician (Maust et al., 2020). In 2019, a Dutch online survey assessed the knowledge of dementia-related risk and protective factors and showed that 56% were unaware of the relation between lifestyle and dementia risk (Heger et al., 2019). In 2022, Barak and colleagues found that a representative sample of older adults from New Zealand were only able to identify 6 out of 14 modifiable dementia risk factors, testifying a low brain health literacy (Barak et al., 2022). *Pdp* participants showed a high level of agreement (88.7%) in response to the closed-ended question 9 (“I found the discussion of my risk factors helpful.”), underlying that this transfer of knowledge about dementia-related risk factors has been achieved. However, the *pdp* is not only successfully addressing the necessity and the need to increase the literacy of dementia-related risk factors, but the programme also supports its participants in cultivating a healthy lifestyle and encourages them to take action to influence their own risk through participation in adapted lifestyle interventions.

The results of the current study reveal that the perceived benefit of the programme is rated slightly lower than the experience of participating in the programme. This encourages a closer examination of intrinsic motivational factors, extrinsic factors and demographic characteristics as proposed by Simone and colleagues (2025). The authors identified four subgroups that differ in their intrinsic factors and further analysed the differences between the subgroups in terms of demographics, extrinsic factors and healthy lifestyle behaviour. They propose that the differences observed in these identified profiles may lead to differences in benefiting from intervention. For instance, a profile characterised by high motivation and low knowledge about dementia will particularly benefit from interventions aimed at psycho-educating about dementia risk. As our results show that most participants are satisfied with the risk communication and education provided in the *pdp*, this approach could identify participants who might benefit from more education/information, and others who may require more practical help in implementing lifestyle changes or need more regular incentives. Identifying such profiles in the *pdp* would not only increase participants’ satisfaction, but also their adherence to interventions and ultimately lead to healthier lifestyle choices. Further, targeting the different subgroups would allow for a more efficient allocation of resources.

Rosenberg et al. (2020) found that knowledge about causes of and risk factors for cognitive disorders is limited and superficial in their subjects. This lack of knowledge was accompanied by stigma, evoking fear, concern, hopelessness and misery in the interviewees. They expressed the need for up to date and reliable information and advice. Our study gives participants the opportunity to ask questions and thus challenge false assumptions about neurocognitive diseases. Furthermore, the programme represents a trustworthy authority for evidence-based support on the topic of dementia prevention. The participants emphasised this competence in 43 free-text comments referring to the professionalism of the team as well as 12 comments stating they felt reassured after participating. 30 participants appreciated having the *pdp* as a point of contact.

A majority of respondents emphasised the good organisation and communication in the programme. As the topic of dementia is strongly associated with fear, shame and stigma, it is particularly important that controllable barriers are kept to a minimum. Therefore, it is crucial that the communication and organisation of such a programme is well structured and straightforward. This also ensures the participation of older or cognitively impaired individuals. To further advance in the development of the *pdp* and ensure the quality of the risk factor interventions, the team has started to take action to find solutions to the concerns participants mentioned in the survey. The implementation of the feedback is an active and ongoing pursuit of the *pdp*, which will be expanded further in the future.

### 4.1 Limitations

Despite the respectable response rate, a non-response bias may be present. Our target population consists of individuals with SCD or MCI, a group characterised by elevated levels of neuropsychiatric symptoms such as depression, apathy, or anxiety (Sannemann et al., 2020). This may be a reason for not responding to the survey. Furthermore, we cannot rule out the possibility that the non-responders may be dissatisfied with the programme. Since the survey ensured participant anonymity, it does not allow conclusions to be drawn on differences in participants’ demographics or their neuropsychiatric symptoms depending on their answers in the survey. However, this loss of information was accepted for the sake of ensuring truthful answers to the questionnaire as literature has shown that people answer more honestly when their anonymity is preserved (Stantcheva, 2023). Furthermore, it was not recorded whether the participants completed the questionnaire alone or with the help of a relative. A recall bias may be present for some participants in Phase 1, due to the long interval between participation in the *pdp* and the survey, which is reflected by the lower response rate for Code 1 participants. In addition, the fact that the sample population is characterised by cognitive deficits should be kept in mind, as some participants may have had difficulties to recall their experiences due to cognitive impairment. Considering response bias in general, the mean should serve as a point of reference and less weight should be given to individual ranking. As there is a strong variance in the cognitive performance of the participants and in the response interval, these variables should not influence the overall results.

In order to examine programme-specific aspects, we designed a new survey instead of using existing generic or disease-specific instruments. Thus, no psychometric quality criteria to our questionnaire are available, as no validity or reliability check was done. In addition, the broad phrasing of the free text questions led to a wide range of answers from participants, but may have impacted the representativeness and validity (Corner et al., 2013). Topics and subtopics have emerged from the qualitative data based on an inductive approach and were confirmed by peer feedback, although the topics cannot be claimed to be exhaustive.

### 4.2 Outlook

The feedback received from the satisfaction survey, especially concerning improvement, will continue to be a priority for future adjustments and help to further increase the quality of the programme. In the future, *pdp* is to be expanded by a participant advisory board. This board will facilitate the incorporation of participants’ perspectives into planned changes to the programme from the start. As accessibility and mobility were often identified as barriers to participation in interventions, high-income countries should utilise monetary resources to remove these barriers. This can be achieved through a dense network of services, but also by ensuring that they are easily accessible via public transport.

The survey results imply that certain constructs, such as the reduction of dementia stigma, or the question on how the communication of risk factors and neuropsychological test results can be improved in at-risk individuals, should be investigated in more detail in the future.

While dementia is still stigmatised, at-risk individuals are interested in learning more about neurodegenerative diseases and healthy ageing. Survey respondents showed a high appreciation for the implementation of a dementia prevention programme and the majority would recommend the *pdp* to others. Programmes such as the *pdp* can promote awareness of dementia-related risk factors and thus promote early action to prevent cognitive decline. This not only helps to reduce the burden on society caused by dementia, but ultimately promotes a self-determined and well-informed way of dealing with the risk of dementia.

## Data Availability

The data supporting the findings of this study are available on request from the corresponding author (dorothee.erz@lih.lu).

## Nomenclature

CNER: Comité National d'Ethique de Recherche
LIBRA: LIfestyle for BRAin health
NPHA: National Poll on Healthy Aging
MCI: mild cognitive impairment
*pdp*: programme dementia prevention
SCD: subjective cognitive decline
SD: standard deviation
