# Supplementary material for: Dementia prevention through the eyes of individuals at risk: insights from a satisfaction survey within the programme for dementia prevention in Luxembourg
Source: Front Aging. 2026 Jan 16;7:1712500. doi: 10.3389/fragi.2026.1712500 (PMC12855403; doi:10.3389/fragi.2026.1712500)
Supplement: Supplementary file 1 [file DataSheet2.pdf]

FEEDBACK QUESTIONNAIRE ON YOUR PARTICIPATION IN THE PROGRAMME FOR DEMENTIA PREVENTION

pdp programm demenz prävention

Dear *pdp* participant,

We are interested in learning how satisfied you were with your participation in the *pdp,* as we would of course like to continuously improve. Therefore, we would be delighted if you could give us some brief, anonymous feedback on your experience with the *pdp*.

We kindly ask for you to send us your feedback **within the next two weeks**.

Please complete the questionnaire below and return it in the enclosed envelope.

or

Complete the questionnaire **online**.

Just scan this QR-code and enter the 4-digit anonymised code:

**Please tick the answer that applies:**	strongly disagree	somewhat disagree	neutral	somewhat agree	strongly agree
1. The team was friendly.					
2. The team was professional.					
3. I felt comfortable.					
4. The waiting time for an appointment was appropriate.					
5. I was satisfied with the communication and the organisation of the appointments by the administrative office.					
6. The waiting time on site was acceptable.					
7. The length of the neuropsychological testing was appropriate.					
8. The participation helped me gain a better understanding of my cognitive performance (e.g. memory, language, concentration, ...).					
9. I found the discussion of my risk factors helpful.					
10. I was satisfied with the time devoted to me in the *pdp*.					
11. I have benefited from the participation.					
12. I would recommend the programme to others.					

uni.lu LCSB

LE GOUVERNEMENT DU GRAND-DUCHÉ DE LUXEMBOURG
Ministère de la Santé

Lieux de consultation:
Maison Médicale Val Fleuri
Maison Médicale Ettelbruck & Belval
Clinique Ste Marie

Tel: (+352) 247-75600
info@pdp.lu
Centre Hospitalier de Luxembourg
23, Val Fleuri, L-1526

pdp programm demenz prävention

Below you have the opportunity to give us your personal feedback on the programme:

1. What did you particularly like about the *pdp*?

2. What parts of our programme could further be improved?

3. Tell us more…
Do you have a personal anecdote about the *pdp*?

Thank you for your participation and see you soon at *pdp*!

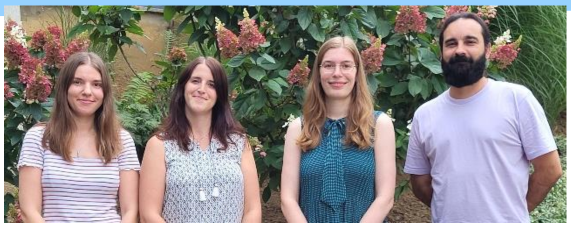

LE GOUVERNEMENT
DU GRAND-DUCHÉ DE LUXEMBOURG
Ministère de la Santé

Lieux de consultation:
Maison Médicale Val Fleuri
Maison Médicale Ettelbruck & Belval
Clinique Ste Marie

Tel: (+352) 247-75600
info@pdp.lu
Centre Hospitalier de Luxembourg
23, Val Fleuri, L-1526
