# Supplementary material for: Dementia prevention through the eyes of individuals at risk: insights from a satisfaction survey within the programme for dementia prevention in Luxembourg
Source: Front Aging. 2026 Jan 16;7:1712500. doi: 10.3389/fragi.2026.1712500 (PMC12855403; doi:10.3389/fragi.2026.1712500)

QUESTIONNAIRE DE FEEDBACK SUR VOTRE PARTICIPATION AU PROGRAMME DEMENCE PREVENTION

pdp programme démence prévention

Cher participant du *pdp*, chère participante du *pdp*,

Nous sommes intéressés à savoir si vous avez été satisfait(e) de votre participation au *pdp,* puisque nous souhaitons évidemment nous améliorer en permanence. C'est pourquoi nous vous serions reconnaissants si vous pourriez nous donner un bref feedback anonyme sur votre expérience avec le *pdp*.

Veuillez nous renvoyer le feedback dans les **deux semaines à venir**.

Pour ce faire, veuillez remplir le questionnaire ci-dessous et nous le renvoyer dans l'enveloppe ci-jointe.

ou

Remplissez le questionnaire **en ligne**.

Pour ce faire, scannez ce code QR et indiquez le code anonymisé composé de 4 chiffres:

**Veuillez cocher la réponse qui convient :**	pas du tout d'accord	plutôt pas d'accord	neutre	plutôt d'accord	tout à fait d'accord
1. L'équipe était sympathique.					
2. L'équipe était professionnelle.					
3. Je me suis senti(e) à l'aise.					
4. Le délai d'attente pour un rendez-vous était acceptable.					
5. J'ai été satisfait(e) de la communication et de l'organisation des rendez-vous de la part du secrétariat.					
6. Le délai d'attente sur place était acceptable.					
7. La durée du test neuropsychologique était appropriée.					
8. La participation m'a aidé à mieux comprendre ma performance cognitive (par ex. mémoire, langage, concentration, ...).					
9. J'ai trouvé la discussion sur mes facteurs de risque utile.					
10. J'étais satisfait(e) du temps qui m'a été consacré au *pdp*.					
11. J'ai bénéficié de la participation.					
12. Je recommanderais ce programme à d'autres personnes.					

Tel: (+352) 247-75600
info@pdp.lu
Centre Hospitalier de Luxembourg
23, Val Fleuri, L-1526

Ensuite, vous avez la possibilité de nous faire parvenir vos commentaires personnels sur le programme :

1. **Qu'est-ce qui vous a particulièrement plu dans le *pdp* ?**

2. **Où voyez-vous des possibilités d'amélioration dans notre programme ?**

3. **Racontez-nous plus...**
   Avez-vous une anecdote personnelle à raconter sur le *pdp* ?

Merci beaucoup pour votre participation et à bientôt au *pdp* !

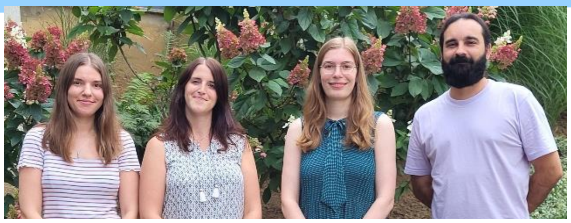

Supplement: Supplementary file 2 [file DataSheet3.pdf]
