# Supplementary material for: Dementia prevention through the eyes of individuals at risk: insights from a satisfaction survey within the programme for dementia prevention in Luxembourg
Source: Front Aging. 2026 Jan 16;7:1712500. doi: 10.3389/fragi.2026.1712500 (PMC12855403; doi:10.3389/fragi.2026.1712500)

pdp programm demenz prävention

FEEDBACK-FRAGEBOGEN ZU IHRER TEILNAHME AM PROGRAMM DEMENZ PRÄVENTION

Lieber *pdp*-Teilnehmer, liebe *pdp*-Teilnehmerin,

Wir sind daran interessiert zu erfahren, wie zufrieden Sie mit Ihrer Teilnahme am *pdp* waren, da wir uns selbstverständlich stetig verbessern möchten. Daher würden wir uns freuen, wenn Sie uns ein kurzes, anonymes Feedback zu Ihrer Erfahrung mit dem *pdp* geben.

Wir würden Sie bitten, uns Ihr Feedback innerhalb der **nächsten zwei Wochen** zukommen zu lassen.

Bitte füllen Sie dazu den untenstehenden Fragebogen aus und schicken Sie diesen im beiliegenden Umschlag an uns zurück.

oder

Füllen Sie den Fragebogen **online** aus.

Scannen Sie hierzu diesen QR-Code und geben Sie den 4-stelligen, anonymisierten Code ein:

**Bitte kreuzen Sie die zutreffende Antwort an:**	Stimme überhaupt nicht zu	Stimme eher nicht zu	Neutral	Stimme eher zu	Stimme voll und ganz zu
1. Das Team war freundlich.					
2. Das Team war professionell.					
3. Ich habe mich wohlgefühlt.					
4. Die Wartezeit auf einen Termin war in Ordnung.					
5. Mit der Kommunikation und der Organisation der Termine durch das Sekretariat war ich zufrieden.					
6. Die Wartezeit vor Ort war in Ordnung.					
7. Die Dauer der neuropsychologischen Testung war angemessen.					
8. Die Teilnahme hat mir geholfen ein besseres Verständnis von meiner kognitiven Leistung (z.B. Gedächtnis, Sprache, Konzentration, …) zu bekommen.					
9. Die Besprechung meiner Risikofaktoren fand ich hilfreich.					
10. Mit der Zeit, die mir im *pdp* gewidmet wurde, war ich zufrieden.					
11. Ich habe von der Teilnahme profitiert.					
12. Ich würde das Programm weiterempfehlen.					

Tel: (+352) 247-75600
info@pdp.lu
Centre Hospitalier de Luxembourg
23, Val Fleuri, L-1526

Im Folgenden haben Sie die Möglichkeit uns Ihr persönliches Feedback zum Programm zukommen zu lassen:

1. **Was hat Ihnen besonders gut am *pdp* gefallen?**

2. **Wo sehen Sie für unser Programm noch Verbesserungsbedarf?**

3. **Erzählen Sie uns mehr…**
   Haben Sie eine persönliche Anekdote zum *pdp*?

Vielen Dank für Ihre Teilnahme und bis bald beim *pdp*!

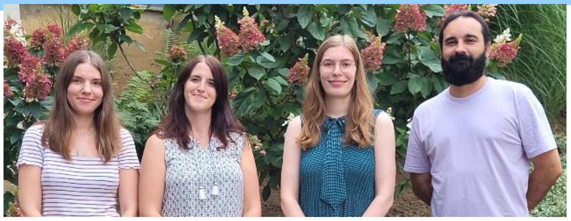

Supplement: Supplementary file 3 [file DataSheet1.pdf]
